# Intranasal Application of Foxp3 Introduced with Poly(d,l-lactic-*co*-glycolic acid) (PLGA) Nanoparticles (Foxp3 NPs) Attenuates Allergic Inflammation in a Mouse Model of Allergic Rhinitis

**DOI:** 10.3390/pharmaceutics17050575

**Published:** 2025-04-27

**Authors:** Seung Cheol Han, Sunhee Yeon, Hyejeen Kim, Sookyoung Park

**Affiliations:** 1 Department of Otorhinolaryngology-Head and Neck Surgery, Chungnam National University Sejong Hospital, Sejong 30099, Republic of Koreadigginonu@cnuh.co.kr (H.K.); 2 SKINMED R&D Center, Daejeon 34037, Republic of Korea; shyeon@skinmed.biz

**Keywords:** allergic rhinitis, FOXP3, microglia, nasal mucosa, PLGA nanoparticle

## Abstract

**Background**: Allergic rhinitis (AR) is a common disease that requires more convenient, safe, and effective therapy. This study aimed to investigate the therapeutic effect of Forkhead box protein3 (Foxp3) introduced with poly(d,l-lactic-*co*-glycolic acid) (PLGA) nanoparticles (Foxp3 NPs) in an AR mouse model. **Methods**: A murine model of allergic rhinitis was established using BALB/c mice through initial sensitization by intraperitoneal administration of ovalbumin (OVA), followed by repeated intranasal OVA challenges. Foxp3 plasmid-loaded PLGA nanoparticles were subsequently administered via either the intranasal or intraperitoneal route to evaluate therapeutic efficacy. Episodes of sneezing and nose rubbing were counted. The serum total IgE, OVA-specific IgE, and cytokine levels in nasal lavage fluid (NALF) were determined by ELISA (Enzyme-Linked ImmunoSorbent Assay). Nasal mucosa from each group were analyzed using protein, reverse transcriptase–polymerase chain reaction (RT-PCR), and histological analyses. **Result**: Rubbing and sneezing symptoms improved in the Foxp3 NPs intranasal administration group. Foxp3 NPs intranasal administration markedly ameliorated OVA-induced nasal allergic inflammation. The total IgE and OVA-specific IgE serum level and IL-4, IL-13 expression levels of NALF were significantly decreased in the treated Foxp3 NPs group. The histopathological results of nasal mucosa were also normal, with no cellular infiltration and no inflammation in the Foxp3 NPs group. **Conclusions**: These results suggest that Foxp3 NPs alleviate nasal allergic inflammation and may have therapeutic value in the treatment of AR.

## 1. Introduction

Allergic rhinitis (AR) is a prevalent inflammatory condition of the nasal mucosa, characterized by symptoms such as sneezing, nasal congestion, rhinorrhea, and nasal itching. AR affects millions of individuals worldwide, significantly impacting their quality of life and contributing to healthcare costs [1,2]. The pathophysiology of allergic rhinitis involves a complex interplay of innate and adaptive immune responses, including innate lymphoid cells, T helper type 2 cells (Th2), eosinophils, immunoglobulin E (IgE), B cells, and mast cells. The inflammation cascade induced by allergens is balanced by allergen tolerance, which is mediated by anti-inflammatory cells, including regulatory T cells (Tregs). Tregs suppress Type1 and Type2 reactions and inhibit allergic responses. Tregs express Forkhead box protein 3 (Foxp3) and CD25 [3]. Foxp3 is critical for the development and function of Tregs [4,5]. In other words, Foxp3 is the master transcription factor essential for the development and function of Tregs [6]. Recent studies have suggested a potential relationship between Foxp3 expression dynamics and allergic rhinitis. Yang et al. reported that luteolin modulates the Treg/Th17 balance to attenuate AR symptoms [7]. Similarly, Su et al. demonstrated effective cytokine suppression using intranasal delivery of miRNA-146a via PEG-PLA hydrogels [8].

Various murine models of allergic rhinitis have been reported using different therapeutic agents and delivery routes. Oral administration was used to evaluate *Asarum heterotropoides* [9], luteolin [7], and quercetin [10], showing immunomodulatory effects. DNA vaccination with Der p2–A20 [11] and herbal treatment [12] also demonstrated efficacy in modulating allergic inflammation. Recent studies have explored nanoparticle-based approaches, including CCL2 siRNA [13], peptide-conjugated alumina nanoparticles [14], and miRNA-146a-loaded hydrogels [8], delivered via intranasal or systemic routes. These diverse models support the rationale for testing Foxp3-encapsulated PLGA nanoparticles as a targeted therapy in the present study. Diverse methods are employed to administer treatments in these models, including oral administration [9,12], intraperitoneal injections, and nanoparticle delivery [11,13,14].

Nanoparticles have been utilized as carriers in several studies to deliver proteins or nucleic acids to regulate allergic rhinitis, primarily in mouse models [8,13,14]. This approach offers the advantage of stable transportation of therapeutic agents into the body. Many proteins were tried to find the effect of introducing proteins or nucleic acids to the ovalbumin-induced AR, such as *Helicobacter pylori* neutrophil-activating protein [14], mucus adhesion promoting protein [14], CCL2 siRNA [13], and miRNA-146a [8]. Nanomedicine-mediated prevention of inflammation response seemed to attenuate the degree of ovalbumin-induced AR in mouse models.

Poly(d,l-lactic-*co*-glycolic acid) (PLGA) is authorized by the Food and Drug Administration (FDA) as a method for targeted delivery of siRNA [15]. PLGA has been successfully utilized as a carrier for pain management and various pharmaceuticals [16]. Notably, PLGA possesses several characteristics that make it suitable for siRNA transport. Initially, its small size facilitates effective penetration of tissues and cellular absorption. Additionally, PLGA nanoparticles provide an effective encapsulation matrix that protects siRNA from enzymatic degradation by RNases, thereby enhancing the stability of siRNA in biological environments [17]. Lastly, the degradation rates of PLGA particles are highly adjustable and can be tailored, allowing for the controlled release of drugs based on the molecular weight of the target and the composition and structure of the nanoparticles for the treatment of pain and for the delivery of certain drugs [18].

In this study, the therapeutic impact of increased Foxp3 expression in an AR was investigated using a mouse model. Foxp3 was introduced into the established BALB/c AR mouse model by encapsulating it in PLGA nanoparticles. This approach allowed us to analyze the effects of Foxp3 delivered via poly(d,l-lactic-*co*-glycolic acid) (PLGA) nanoparticles (Foxp3 NPs) in the AR mouse model [17].

## 2. Materials and Methods

### 2.1. Reagents

Foxp3 NPs were purchased from NanoGlia (NanoGlia, Daejeon, Republic of Korea). Purchased PLGA nanoparticles from the Nanoglia Company were prepared as reported previously, with minor modifications [19]. OVA-specific IgG1, IL-4, and IL10 ELISA kits were purchased from the R&D System (Minneapolis, MN, USA). OVA-specific IgE and total IgE ELISA kits were purchased from MyBioSource (San Diego, CA, USA). Trichrome stain kit, Picro Sirius Red stain kit, and Periodic Acid Schiff (PAS) stain kit were purchased from Abcam (Cambridge, MA, USA). Anti-Foxp3 Alexa fluor 488, Foxp3 Alexa fluor 680 and CD4 Alexa fluor 488 were obtained from eBioscience (San Diego, CA, USA).

### 2.2. Experimental Animals

In total, 70 female BALB/c mice (6–8 weeks old) were purchased from ORIENTBIO Laboratory (Sungnam, Republic of Korea) and were housed under pathogen-free conditions. All animal experiments were approved by the Institutional Animal Care and Use Committee of Chungnam National University and were performed under institutional guidelines (IACUC No. CNUH-020-A0047).

### 2.3. Schematic Flow of Mouse Model

**Figure 1:**
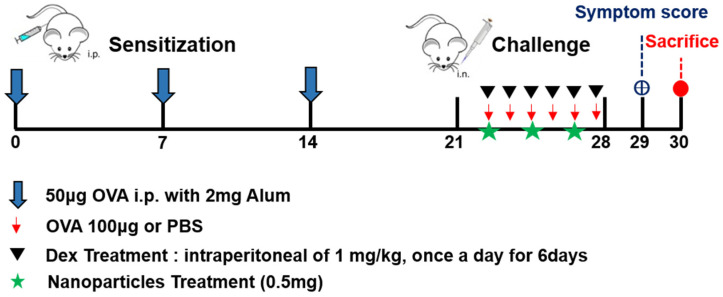
Schematic flow of our experiment. An allergic rhinitis BALB/c mouse model was established through intraperitoneal sensitization with ovalbumin (OVA) followed by intranasal challenge with OVA. The encapsulation of PLGA nanoparticles induced Foxp3. Symptom score was counted before the scarification.

Sensitization of BALB/c mice (excluding the control group) was carried out by intraperitoneal injection of 50 µg OVA mixed with 20 mg/mL aluminum hydroxide in saline, delivered in total volume of 0.2 mL. This procedure was repeated on days 0, 7, and 14 to induce allergic sensitization. This sensitization and challenge protocol has been widely adopted in previous studies of allergic rhinitis mouse models [7]. Then, sensitized mice were randomized into six groups, including OVA group (AR group), Dex group (Dexamethasone treated group, positive control group), Foxp3-I.V group (Foxp3 treated via intraperitoneal injection group), Foxp3-I.N group (Foxp3 treated via intranasal group), Vector-I.V group (intraperitoneal injection negative control group), and Vector-I.N group (intranasal negative control group). Each group’s mice number was 5 or 6. Every six days from day 21 (day 22, 23, 24, 25, 26, 27), all the 6 sensitized mice groups underwent intranasal OVA challenge (100 μg). However, during day 22~day 27, each 6 sensitized mice group was treated differently. OVA group mice (*n* = 10) were treated by sterile normal saline intraperitoneal injection every day, 1 h before the OVA challenge for 6 days. Dex group (*n* = 10) mice were treated with 1 mg/kg Dexamethasone intraperitoneal injection daily, also 1 h before the OVA challenge for 6 days. In the Foxp3-I.V group (*n* = 10) and Foxp3-I.N group (*n* = 10) mice, 1 μg pAAV-EF1a-mFoxp3-T2A-mCherry (pFoxp3) plasmid was intraperitoneally or intranasally injected 2 h before the OVA challenge every 2 days (total of 3 treatments each). In Vector-I.V group (*n* = 10) and Vector-I.N group (*n* = 10) mice, 1 μg pAAV-EF1a-MCS-T2A-mCherry (pNC) plasmid was intraperitoneally or intranasally injected 2 h before the OVA challenge every 2 days (total of 3 treatment each). The Foxp3 NPs dose (1 μg per administration) was determined based on preliminary titration experiments, in which doses between 0.25 μg and 2 μg were evaluated. The 1 μg dose was selected as it yielded consistent immunological effects with minimal inter-animal variability. Control group mice (*n* = 10) were not sensitized but treated with normal saline by intraperitoneal injection and challenged with normal saline. Twenty-four h after the last challenge (day 29), symptom scores were counted for all the mice, and mice were all sacrificed on day 30 (Figure 1). Total IgE, OVA-specific IgE in serum, and cytokine levels in nasal lavage fluid (NALF) were measured. Nasal mucosa was used for protein, reverse transcriptase–polymerase chain reaction (RT-PCR), and histological analyses.

### 2.4. Symptom Scores

The number of sneezing and nasal rubbing instances during 10 min was recorded 24 h after the last challenge and compared between the mice groups.

### 2.5. Histology

Following euthanasia, the entire mouse heads were immersed in 10% neutral buffered formalin for tissue preservation. Subsequently, they underwent decalcification using 1M EDTA at pH 8.0 for 21 days at ambient temperature. The heads were trimmed, embedded in paraffin, and sectioned at a thickness of 4 μm. The sections from each animal were stained with hematoxylin and eosin (H&E) for epithelial thickness, Sirius red for eosinophils, Periodic acid-Schiff (PAS) for goblet cells, and Masson’s trichrome for analysis of collagen deposition. All staining procedures were performed in accordance with the manufacturer’s protocol. Images were obtained using a microscope (BX43, Olympus Corporation, Tokyo, Japan). Epithelial thickness analysis was randomly selected using a high power field of 400× magnification. Positively stained areas of collagen in the mucosa tissues were quantified using high-resolution images by image analysis, using a color-recognition algorithm, in Image J software (version 1.48v; National Institutes of Health, Bethesda, MD, USA). The results were expressed as the percentage of collagen area compared to the total measured area. Finally, the number of eosinophils and goblet cells was calculated by counting the number of positive cells at 400× magnification.

### 2.6. Immunofluorescence Studies

Deparaffinization of the embedded tissue slides was performed using xylene, and rehydration was carried out through a stepwise gradient of ethanol solutions (100%, 95%, and 70% *v*/*v*). Heat-induced antigen retrieval was conducted at 120 °C in a Decloaking Chamber (Biocare Medical, Concord, CA, USA) to restore epitope accessibility. To inhibit non-specific protein binding, a protein-blocking solution composed of 10% normal chicken serum (Vector, Burlingame, CA, USA) in phosphate-buffered saline (PBS) containing 0.3% Triton X-100 (Biosesang, Seongnam, Republic of Korea) was applied to the tissues for 1 h at room temperature. Sections were then incubated in a 1% hydrogen peroxide solution (Sigma-Aldrich, St. Louis, MO, USA) prepared in PBS with 0.3% Triton X-100 for 30 min at room temperature to block endogenous peroxidase activity [20]. Subsequently, tissues were incubated overnight at 4 °C with primary antibodies including anti-CD4-Alexa Fluor 488 antibody, anti-Foxp3-Alexa Fluor 680 antibody, and Foxp3-Alexa Fluor 488 antibody (eBioscience, San Diego, CA, USA). Following washing steps with PBS, nuclear counterstaining was performed using 300 nM 4′,6-diamidino-2-phenylindole (DAPI; Invitrogen, Carlsbad, CA, USA). After a final rinse with PBS, tissue samples were mounted with Fluro-Gel containing Tris Buffer (Electron Microscopy Science, Hatfield, PA, USA) [20]. Confocal images were then captured using a Leica TCS SP8 confocal microscope (Olympus, Tokyo, Japan). The number of CD4/Foxp3 double-positive cells was quantified in three randomly selected high-powered fields (HPF, ×400 magnification) per tissue sample, and mean values with ranges were calculated.

### 2.7. RT-PCR

Total RNA was extracted from nasal tissue samples using TRIzol™ reagent (Invitrogen, Carlsbad, CA, USA), following the standard protocol provided by the manufacturer. cDNA synthesis was carried out using 1 µg RNA and a commercially available reverse transcriptase kit (SuperScript™ III) (Invitrogen, Carlsbad, CA, USA). For cDNA synthesis, 1 μg of total RNA was transcribed with SuperScriptTM III Reverse Transcriptase (Invitrogen, Carlsbad, CA, USA), according to the manufacturer’s instructions. PCR was performed for cDNA synthesis using a T100TM Thermal Cycler (Bio-Rad Laboratories, Hercules, CA, USA). The mRNA expression was analyzed using a CFX Connect TM Real-Time PCR Detection System (Bio-Rad Laboratories, Hercules, CA, USA) with PowerUp TM SYBR TM Green Master Mix (Applied Biosystems, Carlsbad, CA, USA). This approach to quantify cytokine expression using SYBR Green qPCR has been utilized in similar allergic rhinitis studies [10]. The primers used are presented in Table 1. All PCR assays were performed in triplicate. For each sample, the differences in threshold cycles between the target genes and GAPDH were determined as follows:

ΔC_T = C_T^{target gene} − C_T^{reference gene}(1)

ΔΔC_T = ΔC_T^{sample} − ΔC_T^{control}(2)

RQ = 2^{−ΔΔC_T}(3)

### 2.8. Western Blot Analysis

The mucosa tissues were homogenized with cold RIFA buffer (Cell Signaling Technology, Danvers, MA, USA) containing protease inhibitors (iNtRON Biotechnology, Seongnam, Republic of Korea). The total protein concentration was determined using the Bradford assay (Bio-Rad, Hercules, CA, USA). The samples were resolved in 12% sodium dodecyl sulfate polyacrylamide gel electrophoresis (SDS-PAGE), transferred to 0.45 μm Polyvinyl Difluoride (PVDF) membranes, and then analyzed separately. The membranes were blocked using 5% skim milk solution, and then membranes were incubated with anti-Foxp3 (eBioscience, Ireland, UK). After incubation, the membranes were washed 3 times with TBST buffer (20 mmol/L Tris-buffered saline and 0.1% Tween 20) for 1 h. Peroxidase conjugated anti rat-IgG were used as secondary antibodies. Chemiluminescence was performed with the Amersham ECL plus Western blotting detection system (GE Healthcare, Chicago, IL, USA).

### 2.9. ELISA Assay in the Serum and NALF

Mice blood and NALF were collected from each group after the last challenge. To quantify cytokine levels, nasal lavage fluid was processed by centrifugation at 12,000 rpm for 10 min at 4 °C, and the resulting supernatant was used for ELISA assays. Mice blood was centrifuged for 20 min at 2000 rpm to separate the serum. Levels of cytokines in NALF were measured by Ready-Set-Go mouse IL-4 (R&D System, USA) ELISA, mouse IL-10 ELISA (R&D System, USA), and Ready-Set-Go mouse IL-13 ELISA (Abcam, Cambridge, MA, USA) according to the manufacturer’s instructions, respectively. Quantification of IL-4, IL-10, and IL-13, in NALF by ELISA follows standard procedures used in prior murine AR models [10]. Serum levels of OVA-specific IgE, Total IgE, and OVA-specific IgG1 were detected using an ELISA kit (R&D Systems and MyBioSource, USA) following the manufacturers’ instructions.

### 2.10. Statistical Analysis

Statistical analyses were conducted using SPSS version 22.0.0.0 (International Business Machines, Armonk, NY, USA) and GraphPad Prism version 6.01 (GraphPad Software, La Jolla, CA, USA). Statistical evaluation between two experimental groups was conducted using the Mann–Whitney U test for non-normally distributed data. For comparing multiple groups, Kruskal–Wallis analysis was applied with appropriate post hoc comparisons. Additionally, two-way ANOVA was used for analyzing multiple variables simultaneously. Results are presented as means ± SEM, and statistical significance was set at *p* < 0.05.

## 3. Results

**Figure 2:**
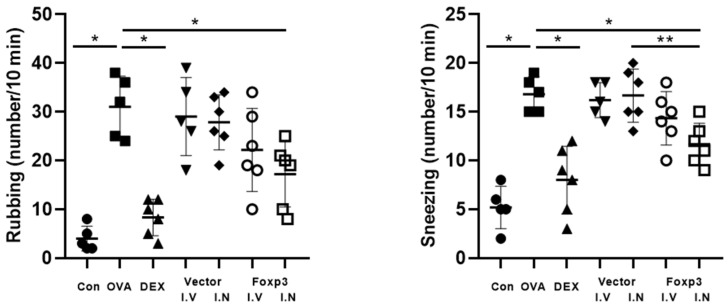
Numbers of nose-rubbing behaviors and sneezes during 10 min were recorded within 30 min after the last challenge and compared between the experimental groups. * *p* < 0.05, ** *p* < 0.001; Dex, positive control group; Foxp3 I.V, I.N, Foxp3 treatment group; Vector I.V, I.N, negative control group; CON, control group; OVA, ovalbumin.

From the comparison of symptom scores, there were significant differences in the numbers of nose-rubbing behaviors and sneezing between the groups. For rubbing behaviors, Dex group and Foxp3-I.N group mice had significantly fewer numbers than OVA group mice. Dex group and Foxp3-I.N group mice also had significantly fewer sneezing numbers than OVA group mice. In addition, Foxp3-I.N group mice had significantly fewer sneezing numbers than Vector-I.N group mice (Figure 2).

**Figure 3:**
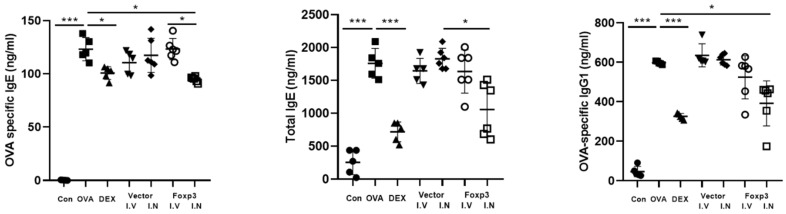
ELISA of the serum of five mice from each group. OVA-specific IgE, Serum total IgE, and OVA-specific IgG1 levels were significantly lower in the Dex and Foxp3 I.N groups. * *p* < 0.05, *** *p* < 0.0001; Dex, positive control group; Foxp3 I.V, I.N, Foxp3 treatment group; Vector I.V, I.N, negative control group; CON, control group; OVA, ovalbumin, ELISA, enzyme-linked immunosorbent assay.

From the comparison of OVA-specific IgE, total IgE, and OVA-specific IgG1, there were also some significant differences between the groups. Foxp3-I.N group mice had significantly lower total IgE than Vector-I.N group mice. Also, Foxp3-I.N group mice had significantly lower OVA-specific IgE and IgG1 than OVA group mice. OVA-specific IgE of Foxp3-I.N group mice were also significantly lower than Foxp3-I.V group. On the other hand, Dex group mice also showed significantly lower total IgE, OVA-specific IgE, and IgG1 than OVA group mice (Figure 3).

**Figure 4:**
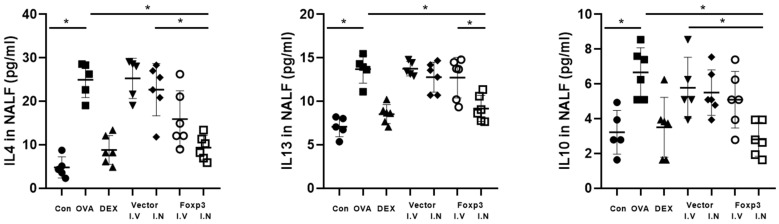
ELISA of the NALF of five mice from each group. NLF concentrations of IL-4, IL-13, and IL-10 were significantly lower in the Dex and Foxp3 I.N groups. * *p* < 0.05; Dex, positive control group; Foxp3 I.V, I.N, Foxp3 treatment group; Vector I.V, I.N, negative control group; CON, control group; OVA, ovalbumin ELISA, enzyme-linked immunosorbent assay; IL, interleukin; NALF, nasal lavage fluid.

From the analysis of ELISA data from NALF, Foxp3-I.N group mice had significantly lower IL-4, 10, and 13 than OVA group mice. Also, IL-13 of Foxp3-I.N group mice were significantly lower than Foxp3-I.V group mice. In addition, IL-4 of Foxp3-I.N group mice were lower than Vector-I.N group mice and IL-10 of Foxp3-I.N group mice were lower than Vector-I.V group mice. On the other hand, Dex group mice did not show any significant differences with other groups (Figure 4).

**Figure 5:**
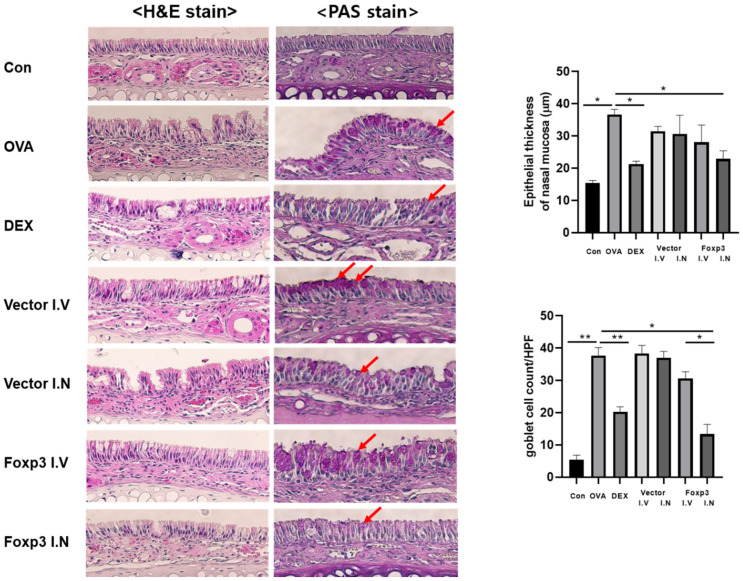
A 4 μm-thick specimen was embedded in paraffin and stained with hematoxylin, eosin (H&E), and periodic acid Schiff (PAS). The thickness of nasal mucosa epithelium was significantly lower in the Dex and Foxp3 I.N groups. Goblet cell counts notably decreased in the Dex and Foxp3 I.N groups. The red arrow shows the increased number of Goblet cells. (original magnification, ×40). * *p* < 0.05, ** *p* < 0.001; Dex, positive control group; Foxp3 I.V, I.N, Foxp3 treatment group; Vector I.V, I.N, negative control group; CON, control group; OVA, ovalbumin.

**Figure 6:**
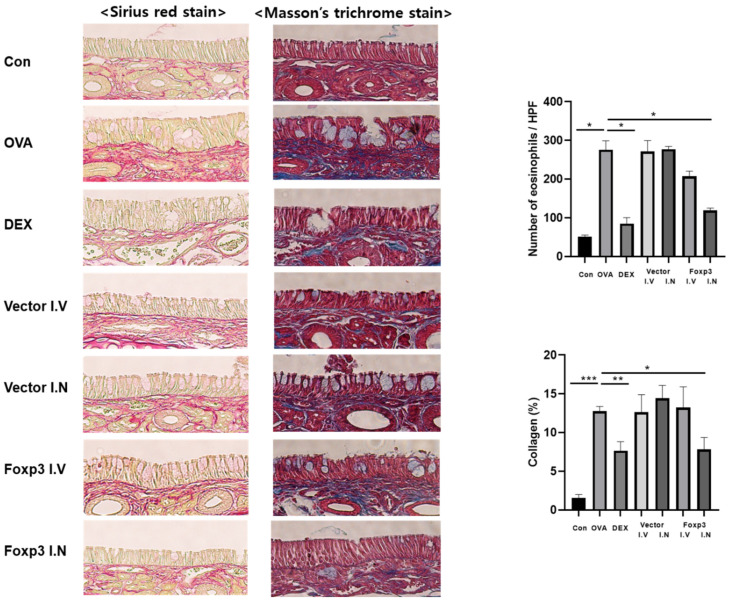
A 4 μm-thick specimen embedded in paraffin and stained with Sirus red and Masson’s trichrome. The number of eosinophils in the nasal mucosa and the percentage of collagen notably decreased in the Dex and Foxp3 I.N groups (original magnification, ×40). * *p* < 0.05, ** *p* < 0.001, *** *p* < 0.0001; Dex, positive control group; Foxp3 I.V, I.N, Foxp3 treatment group; Vector I.V, I.N, negative control group; CON, control group; OVA, ovalbumin.

From the histological analysis of nasal mucosa, the thickness of nasal mucosa epithelium was measured from a 4 μm-thick specimen stained with H&E. The thickness was significantly lower in Foxp3-I.N group mice and Dex mice, compared to OVA group mice. From the PAS stain, the goblet cell count in Foxp3-I.N group mice and Dex mice were also significantly lower than OVA group mice. Foxp3-I.N group mice even had lower goblet cell count than Foxp3-I.V group mice (Figure 5). The number of eosinophils in Foxp3-I.N group mice and Dex mice were significantly lower than OVA group mice from Serus red staining. In addition, the collagen percentage had the same result from Masson’s trichrome staining (Figure 6).

**Figure 7:**
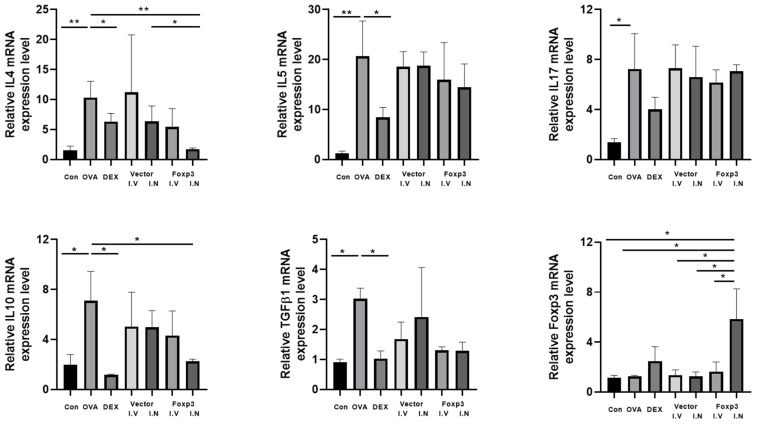
mRNA levels in nasal tissue revealed by RT-PCR. The expression levels of IL-4 and IL-10 were significantly lower in the Dex and Foxp3 I.N groups. However, there were no significant differences in IL-5 levels among the groups * *p* < 0.05, ** *p* < 0.001; Dex, positive control group; Foxp3 I.V, I.N, Foxp3 treatment group; Vector I.V, I.N, negative control group; CON, control group; OVA, ovalbumin; IL, interleukin; mRNA, messenger RNA; RT-PCR, reverse transcriptase–polymerase chain reaction.

There were also some significant results from the analysis of mRNA expression levels in nasal tissue. The expression levels of IL-4 and IL-10 were significantly lower in the Dex and Foxp3 I.N group mice than OVA group mice. However, only Dex group mice showed significantly lower expression levels of mRNA than the OVA group about IL-5 and TGFβ-1 (Figure 7). IL-17 mRNA expression level did not show any significant differences between the groups.

**Figure 8:**
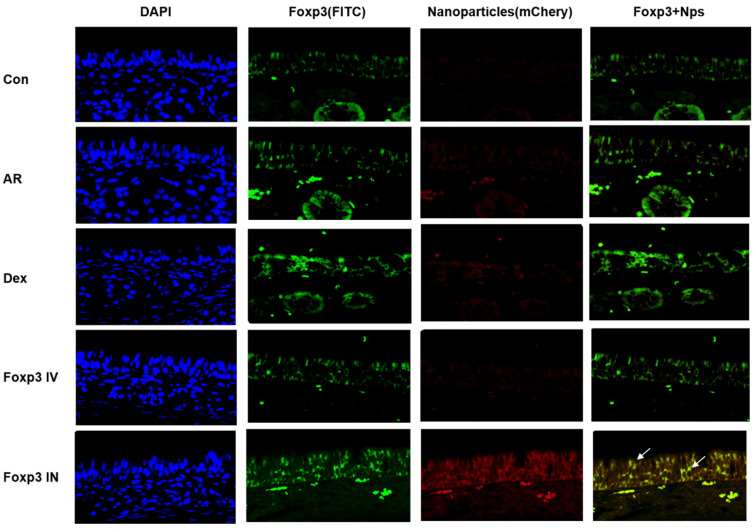
Immunofluorescence for Foxp3 (green) and Nanoparticle (red) co-localization in mucosal epithelial cells. LPS/ATP-induced NLRP3/ASC complexes were inhibited. The scale bar indicates 100 μm. Dex, positive control group; Foxp3 I.V, I.N, Foxp3 treatment group; CON, control group.

Foxp3 NPs expression pattern of nasal mucosa epithelium was examined I.N and I.V group mice using immunofluorescent staining. Stimulation of I.N group increased expression of Foxp3-nanoparticle double-positive cells compared with control, AR. Dex, and I.V group mice (Figure 8, white arrows).

## 4. Discussion

AR is a chronic non-infectious disease mediated by IgE and occurs in allergen-sensitive individuals. The classic symptoms of allergic rhinitis are sneezing, watery rhinorrhea, nose itches, and nasal congestion. The quality of life of patients is seriously affected by allergic rhinitis. In recent years, studies have found that the prevalence rate of allergic rhinitis has increased significantly [21].

Oral or topical antihistamines and corticosteroids are primary treatments that prevent or suppress allergic reactions and immune responses. However, their use is controversial in certain patients, such as those who are pregnant or breastfeeding and individuals with underlying conditions like diabetes [22]. Additionally, commercially available nasal sprays have drawbacks, including common side effects like nasal irritation and epistaxis, the need for frequent administration, and a bitter taste due to the connection between the nose and mouth [23]. Given the limitations of current treatments, developing new methods for managing allergic rhinitis is critical. This study investigated the use of Foxp3 NPs designed to target and suppress allergic reactions and immune responses. Intranasal administration of Foxp3-PLGA nanoparticles was performed 2 h prior to each OVA challenge. This timing was based on prior pharmacokinetic studies reporting that PLGA nanoparticles are efficiently absorbed through nasal mucosa and begin releasing their payload within 1–3 h after delivery [16,17]. Although retention was not directly measured in this study, prior work has demonstrated that PLGA nanoparticles administered intranasally can remain within mucosal cells for up to 72 h, depending on physicochemical properties and mucosal absorption dynamics [17].

The present study compared symptom scores and a range of immunological and histological parameters across different groups of mice. Notably, Dex and Foxp3-I.N groups showed significantly reduced nasal symptoms compared to the other groups. Immunologically, these groups had lower levels of total IgE, OVA-specific IgE, and IgG1 than the other groups. From cytokine level comparisons (from NALF ELISA), the Foxp3-I.N group had lower levels of IL-4, IL-10, and IL-13 than the OVA and Vector groups. Histological analyses indicated thinner nasal mucosa and fewer goblet cells in the Dex and Foxp3-I.N groups compared to the OVA group. Similarly, eosinophil counts and collagen deposition were lower in these groups. Regarding mRNA expression, there was a decrease in IL-4, IL-10, IL-5, and TGFβ-1 in the Dex and Foxp3-I.N groups relative to the OVA group, with no significant changes in IL-17 levels. These results suggest that comparing the curative effects of the control group and treatment group, intranasal Foxp3 NPs have the potential to be a new alternative in the treatment of allergic rhinitis. Interestingly, IL-5 and IL-17 mRNA levels were not significantly reduced in the Foxp3 NP-treated group. This may be due to the pathway-specific nature of Foxp3-mediated immunoregulation, which primarily suppresses Th2 cytokines such as IL-4 and IL-13, while exerting less direct influence on IL-5 and Th17-associated IL-17. Moreover, IL-5 is tightly linked to eosinophil survival, and IL-17 is more characteristic of neutrophilic inflammation, which may not be dominant in this AR model.

Some studies have also utilized nanoparticles and AR mouse models [13,14]. However, the present study possesses strengths compared to previous investigations, as it employed both intranasal (I.N) and intraperitoneal (I.V) administration of nanoparticles. Notably, it not only compared symptom scores, immunoglobulin levels, and cytokine concentrations, but also included a histological analysis—an aspect not addressed in the other two nanoparticle-based AR mouse model studies [13,14]. These strengths enabled a more direct demonstration and analysis of the effects of intranasal injection of Foxp3 NPs on allergic rhinitis in the mouse model. Recent studies have indicated that nasal drug delivery routes provide significant benefits, including the potential bypassing of the first-pass effect and the avoidance of pre-systemic elimination in the gastrointestinal tract. This approach may allow for therapeutic effects to be achieved with lower doses of certain medications [24]. Among nasal delivery methods, intranasal administration is particularly advantageous due to its large absorptive surface area and high vascularity, in addition to encompassing all previously mentioned benefits [25].

The results indicate that the effect of Foxp3 NPs injection was comparable to that of dexamethasone. This result is very understandable because glucocorticosteroids effectively decrease allergic inflammation. Also, from one study [26], the AR mouse model’s decreased number of CD4^+^Foxp3^+^ T-cells when treated with dexamethasone is already shown. Therefore, the observed similarity in the effects of the Dex and Foxp3-I.N groups can be explained by this finding. Given the widespread use of steroid nasal sprays in patients with allergic rhinitis, it is anticipated that Foxp3 NPs nasal sprays may one day become a common therapeutic option for these patients. This study used nanoparticles to deliver Foxp3 by the intranasal route effectively. Nanoparticles are effective carriers suitable for multiple drug administration routes. Both natural and synthetic polymers have been extensively explored to enhance the biocompatibility and biodegradability of nanoparticles. Among these, PLGA, utilized in this research, is frequently selected due to its excellent biocompatibility and its ability to resorb naturally.

The limitation of this study was that only I.N injection of Foxp3 mice showed the effect of decreased AR. And the nanoparticles were not designed and synthesized by us and therefore have limitations in terms of novelty. Another limitation of the present study is the absence of measurements of Th1/Th2 and Treg/Th17 cell ratios, as well as Tet2 and Notch1 expression levels. Additionally, IL-18 levels were not assessed. These immunological parameters could have provided further insights into the mechanisms underlying the therapeutic effects of Foxp3 NPs. And although TGF-β is a key cytokine associated with Treg differentiation and Foxp3 function, it was not measured in this study due to constraints in sample volume and assay scope. Future studies incorporating TGF-β quantification may better elucidate the immunomodulatory pathways involved in Foxp3 NP-mediated regulation. In addition, because the results were induced from mice, other animal models’ studies should be conducted before applying them to human patients. Only female mice were used in the present study, a choice made to control for sex-specific physiological responses due to the limited number of animals available. However, this limitation may have obscured potential differences in outcomes between sexes. Additionally, this study employed only OVA-induced AR mouse models. Therefore, the effects of Foxp3 injection in other types of AR models remain uncertain and cannot be predicted based on the current findings. In other words, the effects of Foxp3 injection in other AR mouse models cannot be determined based on the current study. These represent important limitations, and future investigations will need to include adequate numbers of both male and female mice, as well as explore different types of AR mouse models.

## 5. Conclusions

In conclusion, this study demonstrates that intranasal administration of Foxp3-encapsulated PLGA nanoparticles significantly attenuates allergic inflammation in an AR mouse model. Improvements were confirmed by reduced symptom scores, lower serum total and OVA-specific IgE levels (ELISA), decreased Th2 cytokine expression (RT-PCR), diminished mucosal eosinophilia and goblet cell hyperplasia (histology), and enhanced Foxp3 expression in nasal tissues (immunofluorescence). These findings support the potential of Foxp3 nanoparticles as a targeted immunomodulatory therapy for allergic rhinitis.

## Figures and Tables

**Table 1:** Primer sequences used for reverse transcription–quantitative polymerase chain reaction.

Gene	Sequence (5′-3′)
IL-4	F: ATCATCGGCATTTTGAACGAGGTC R: ACCTTGGAAGCCCTACAGACGA
IL-5	F: GATGAGGCTTCCTGTCCCTACT R: TGACAGGTTTTGGAATAGCATTTCC
IL-10	F: CGGGAAGACAATAACTGCACCC R: CGGTTAGCAGTATGTTGTCCAGC
IL-13	F: AACGGCAGCATGGTATGGAGTG R: TGGGTCCTGTAGATGGCATTGC
TGFβ-1	F: TGATACGCCTGAGTGGCTGTCT R: CACAAGAGCAGTGAGCGCTGAA
Foxp3	F: CCTGGTTGTGAGAAGGTCTTCG R: TGCTCCAGAGACTGCACCACTT
GAPDH	F: CATCACTGCCACCCAGAAGACTG R: ATGCCAGTGAGCTTCCCGTTCAG

Foxp3, Forkhead box protein; F, forward; R, reverse.

## Data Availability

Data are contained within the article.

## Abbreviations

The following abbreviations are used in this manuscript:

ANOVA	Analysis of Variance
AR	Allergic Rhinitis
BALB/c	Bagg Albino Laboratory-Bred/c strain (mouse)
CD4	Cluster of Differentiation 4
CD25	Cluster of Differentiation 25
DAPI	4′,6-Diamidino-2-Phenylindole
EDTA	Ethylenediaminetetraacetic Acid
ELISA	Enzyme-Linked Immunosorbent Assay
Foxp3	Forkhead Box Protein 3
GAPDH	Glyceraldehyde 3-Phosphate Dehydrogenase
HPF	High Power Field
IACUC	Institutional Animal Care and Use Committee
IgE	Immunoglobulin E
IL	Interleukin
NALF	Nasal Lavage Fluid
OVA	Ovalbumin
PAGE	Polyacrylamide Gel Electrophoresis
PAS	Periodic Acid–Schiff
PBS	Phosphate-Buffered Saline
PCR	Polymerase Chain Reaction
PLGA	Poly(d,l-lactic-*co*-glycolic acid)
PVDF	Polyvinylidene Fluoride
qPCR	Quantitative Polymerase Chain Reaction
RNA	Ribonucleic Acid
RT-PCR	Reverse Transcriptase–Polymerase Chain Reaction
SEM	Standard Error of the Mean
SDS	Sodium Dodecyl Sulfate
TBST	Tris-Buffered Saline with Tween 20
